# Two different approaches for pharmacokinetic modeling of exhaled drug concentrations

**DOI:** 10.1038/srep05423

**Published:** 2014-06-24

**Authors:** S. Kreuer, A. Hauschild, T. Fink, J. I. Baumbach, S. Maddula, Th. Volk

**Affiliations:** 1 Department of Anaesthesiology, Intensive Care and Pain Therapy, Saarland University Medical Center and Saarland University Faculty of Medicine, 66482 Homburg/Saar; Germany; 2 Max Planck Institute for Informatics, Research Group on Computational Systems Biology, Campus E2.1, R. 203, 66123 Saarbrücken; Germany; 3 Reutlingen University, Faculty Applied Chemistry, Alteburgstrasse 150, 72762 Reutlingen; Germany; 4 B&S Analytik, BioMedicalCenter Dortmund, Otto-Hahn-Str. 15, 44227 Dortmund; Germany

## Abstract

Online measurement of drug concentrations in patient's breath is a promising approach for individualized dosage. A direct transfer from breath- to blood-concentrations is not possible. Measured exhaled concentrations are following the blood-concentration with a delay in non-steady-state situations. Therefore, it is necessary to integrate the breath-concentration into a pharmacological model. Two different approaches for pharmacokinetic modelling are presented. Usually a 3-compartment model is used for pharmacokinetic calculations of blood concentrations. This 3-compartment model is extended with a 2-compartment model based on the first compartment of the 3-compartment model and a new lung compartment. The second approach is to calculate a time delay of changes in the concentration of the first compartment to describe the lung-concentration. Exemplarily both approaches are used for modelling of exhaled propofol. Based on time series of exhaled propofol measurements using an ion-mobility-spectrometer every minute for 346 min a correlation of calculated plasma and the breath concentration was used for modelling to deliver R^2^ = 0.99 interdependencies. Including the time delay modelling approach the new compartment coefficient k_e0lung_ was calculated to k_e0lung_ = 0.27 min^−1^ with R^2^ = 0.96. The described models are not limited to propofol. They could be used for any kind of drugs, which are measurable in patient's breath.

In medicine, drug dosing is usually adapted to patient's weight. However, the blood concentration of medications varies considerably between different individuals. The blood concentration depends on the liberation, absorption, distribution, metabolism and the excretion of the drug. These factors are influenced by normal variability of the individual, genetic influences particularly in cytochrome P450 system and organ dysfunctions. These relationships are described as pharmacokinetics (PK).

Different compartment^1^ and non-compartment PK models e.g. physiological models^2^ are used to calculate plasma or effect concentrations of drugs. These models are based on normal population PK data sets. However, the concentrations are only calculated and the difference to the real drug blood concentration could be substantially^3^. The measurement of blood concentration e.g. by High-Performance Liquid Chromatography (HPLC) is expensive, complex and has a time delay of several hours. In clinical practise the measurement of blood concentrations is only performed for a few drugs^4^.

Therefore, a new promising approach is the online measurement of drug concentrations in patient's breath^5^. Until now, the number of drugs which were detected in patient's breath is limited: e.g. methadone^6^, propofol^7^, valproic acid^8^.

Actually, different methods are under consideration, including mass spectrometry, ion mobility spectrometry and chemical sensors. In the present paper, we will not focus on the experimental details, conditions, advantages and disadvantages of the different methods. The starting condition is, that such measurements can be carried out and time series of breath concentration values are available within short time intervals. Acceptable would be the range of one value per minute of exhaled breath concentration, with a total analysis time in the same range of 1 minute. Consequently, one value each minute with 1 minute retarding will be considered as provided by an experimental breath analysis method.

However, a direct transfer between breath concentration and blood concentration is not possible and leads to poor correlation values, because the diffusion of drugs through the capillary wall of the alveoli takes time. If the drugs blood concentration increases or decreases the exhaled concentration follows with a time delay^9^.

Classically the diffusion of a drug from blood through the lung into air is described by the blood/gas partition coefficient (Ostwald Coefficient). This coefficient is defined as the ratio of the concentration in blood to the concentration in gas that is in contact with that blood when the partial pressure in both compartments is at equilibrium. It is thus a dimensionless number. The higher the blood/gas partition coefficient, the more soluble is the anaesthetic in blood compared to air and the more it binds to lipids and proteins in the blood. If an anaesthetic has a high Ostwald coefficient, then a large amount of it will have to be taken up in the body's blood before being passed on to the fatty (lipid) tissues of the brain where it can exert its effect^10^.

The blood/gas partition coefficient is widely used for the PK description of volatile anaesthetics^10^. However, for PK modelling the coefficient could only be used if the partial pressure in both compartments is at equilibrium. Depending on the drug it could take several hours to reach this steady state. Therefore, the introduction of breath concentrations measured within time series needs a model to include the concentration values measured in breath without the precondition of compartments within equilibrium.

To overcome the precondition of equilibrium, we would like to demonstrate in this manuscript two different PK modelling approaches to handle the drug breath concentrations. The calculations are performed on the basis of concentration data of the measurements of a patient, who received the intravenous anaesthetic propofol. The propofol breath concentration was measured by a modified ion mobility spectrometer combined with a multi-capillary-column (P-IMS)^11^, which delivers one peak analysis every minute with 1 minute total analysis time including sampling of 10 mL exhaled breath. It should be noted, that the presented models are not specific for propofol. In principle, the mathematical descriptions could also be used for other drugs.

## Methods

### Clinical measurements

With approval of the local Ethics Committee (Ärztekammer des Saarlandes, Saarbrücken, Germany) and written informed consent an adult patient scheduled for an abdominal surgical procedure was studied. The patient received propofol and remifentanil (opioid) as a target controlled infusion (TCI). The concept of TCI is to estimates calculated plasma and effect site concentrations based on pharmacokinetic 3 compartment model^12^.

The breath concentration values were measured using an ion mobility spectrometer type BioScout (B&S Analytik GmbH, Dortmund, Germany), with the internal Multi-capillary-column (MCC) adjusted to 90°C to reach a retention time of propofol on the MCC (type OV-5, Multichrom, Novosibirsk, Russia) of about 20 s. A total volume of 10 mL was taken on the MCC. For further details considering the method see^11^, for the experimental setup see^13^. Samples of exhaled air were automatically taken every minute by the P-IMS.

**Figure 1:**
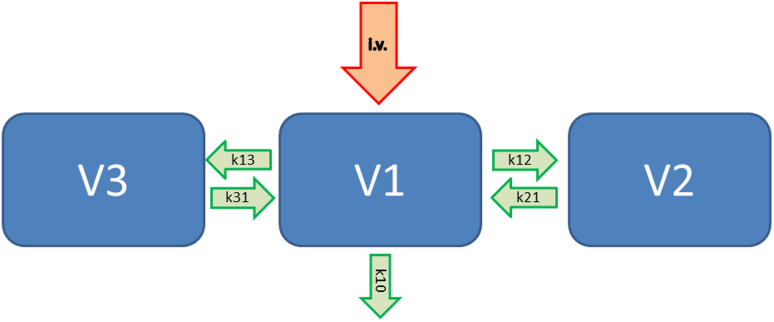
Three compartment pharmacokinetic model. K_XX _ = rate constants for metabolism and elimination, V1 = Volume of central compartment/blood concentration, V2 = V1*k_12_/k_21_, V3 = V1*k_13_/k_31_, Elimination clearance = V1*k_10_, Clearance 2 = V2*k_21_, Clearance 3 = V3*k_31._

A typical pharmacokinetic model including 3 compartments is shown in Figure 1. Here, V1 represents the central blood compartment, the other 2 compartments named V2 and V3 are considered as an additional fast and another slow metabolizing compartment. Therefore, the incoming intravenous drug (i.v.) will go with the reaction coefficient k_10_ out of the body (elimination) and with e.g. k_13_ from V1 to V3 and k_31_ from V3 back to V1, respectively. The clearance of the central compartment is described as V1 * k_10_ and the concentration in compartment V3 is represented by the value V3 = V1 * k_13_/k_31_. Thus, the clearance 2 is V2 * K21.

For the administration of propofol we used the Marsh data set^14^ with a constant target concentration of 3 µg/ml in compartment V1. The corresponding values of V1 and the reaction coefficients are shown in Table 1. Here, following to the Marsh model, V1 is calculated with the weight of the patient multiplied with 0.228 (Table 1).

The major disadvantage of TCI is that the concentrations are only calculated and may have a deviation of about 25%^3^ in healthy patients. The remifentanil target concentration (Minto, plasma mode)^15^ was adjusted to clinical needs.

### Pharmacokinetic modeling

Plotting the exhaled propofol concentration versus the calculated propofol plasma concentration revealed a hysteresis loop (see below). Therefore, it is necessary to include the measured propofol concentration into a pharmacokinetic model.

**Figure 2:**
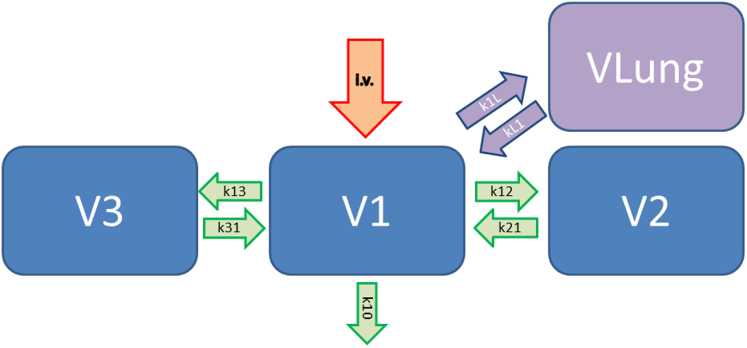
3-compartment pharmacokinetic model extended with an additional lung compartment with a volume (VLung) and clearance (ClLung). LCC = (k1L*PPCO-kL1*LCCO)+LCCO. LCC = Lung compartment concentration, k1L = Cllung/V1, PPCO = Previous Propofol plasma concentration, kL1 = Vlung/Cllung, LCCO = Previous lung compartment concentration, V1 = Volume first compartment marsh model ( = 0,228* patient weight m [kg]). VLung = Volume lung compartment, ClLung = Clearance lung compartment.

Two different approaches for kinetic modelling were tried. First the 3-compartment model was extended. We calculated an additional 2-compartment model based on the first compartment of the Marsh model and a new lung compartment VLung (Fig. 2).

For the relationship between exhaled propofol concentration and the respective propofol lung concentration values a polynomial second order was used. VLung, ClLung and the parameters of the polynomial second order were estimated using nonlinear regression with ordinary least squares.

The computations were performed on a spreadsheet using the Excel 2010 software program (Microsoft). The parameters were optimized to maximise R^2^ using the Solver tool within Excel.

**Figure 3:**
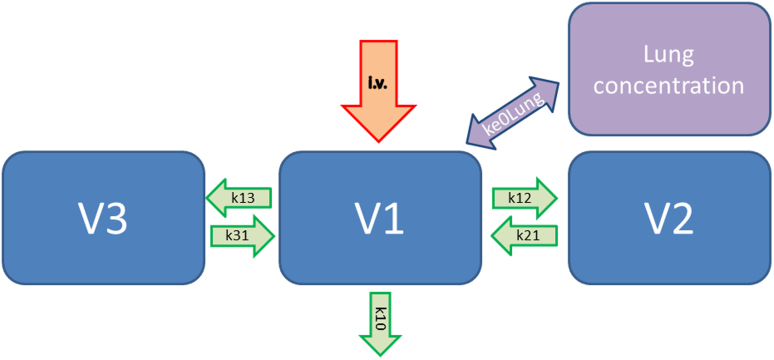
Three compartment pharmacokinetic model the propofol lung concentration is calculated from the blood concentration with a time delay. LCC = (PPC+ (LCCO-PPC))*exp(-k_e0lung_), PPC = Propofol plasma concentration, k_e0lung_ = First-order rate constant determining the equilibration between plasma, and lung concentration.

The second approach was to calculate a time delay of the plasma concentration dependent to the measurements of exhaled propofol concentrations as a first order model (Fig. 3). The computation was performed in the same way as described above.

Concentrations measured with the IMS are given as intensities in volt. Usually concentrations in this range are given as parts per billion [ppb]. The transfer from volt to ppb was performed with a calibration curve. Therefore, the modelling of both approaches was done two times for volt and ppb. A difference occurs due to the non-linear response of the ion mobility spectrometer within the calibration curve.

## Results

**Figure 4:**
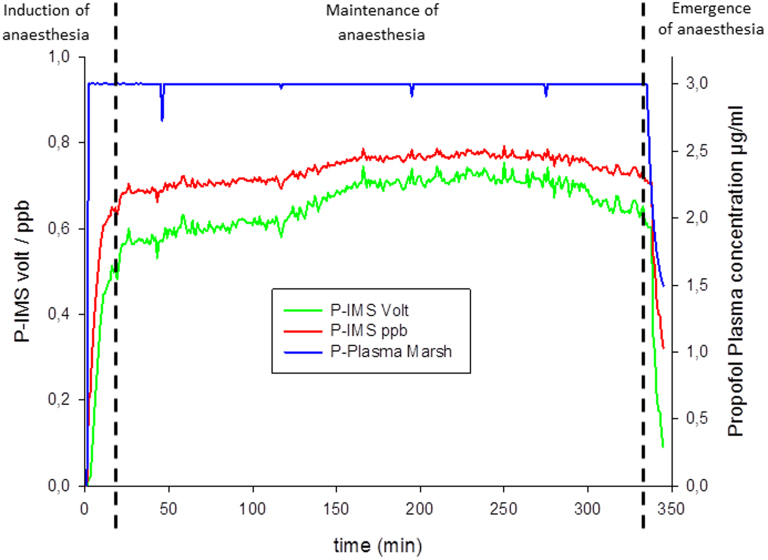
Time course. Time course of the calculated propofol TCI concentration (blue line) and the measured P-IMS breath concentration (red line concentration [ppb], green line peak height [volt]) over 346 min.

The P-IMS was tested in patients under clinical conditions in the operation room. To demonstrate the mathematical PK/PD modeling approaches data from one patient were used. The duration of this measurement was 346 min. We were able to perform one measurement each minute. There was no technical failure of the P-IMS hardware or software. During induction of anaesthesia the exhaled propofol concentration increased with a time delay compared to the TCI plasma concentration. During the maintenance of anaesthesia, the exhaled propofol concentration increased continuously. The propofol TCI target plasma concentration was set stable at 3 µg/ml. At the end of the surgical procedure the propofol TCI administration was stopped. The exhaled propofol concentration decreased with a small time delay (Fig. 4).

### PK-Modeling

**Figure 5:**
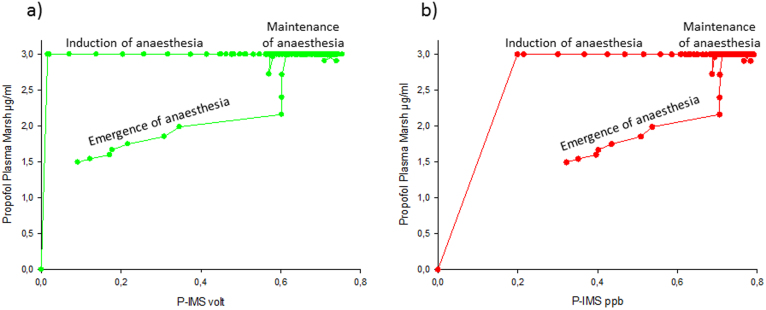
Hysteresis loop between plasma concentration and breath concentration. Calculated propofol plasma concentration versus breath concentration measured by the P-IMS (a) green dots: peak height; [volt]; (b) red dots: concentration [ppb].

Plotting the propofol breath concentration peak intensity [volt] a) or [ppb] b) versus calculated propofol plasma concentration revealed a hysteresis loop (Fig. 5).

Figure 5 indicates that a direct correlation analysis between plasma concentrations and exhaled concentrations is not useful. These calculations lead to poor R^2^ values (R^2^ = 0,32 [volt]; R^2^ = 0,41 [ppb]).

### Propofol lung compartment concentration

**Figure 6:**
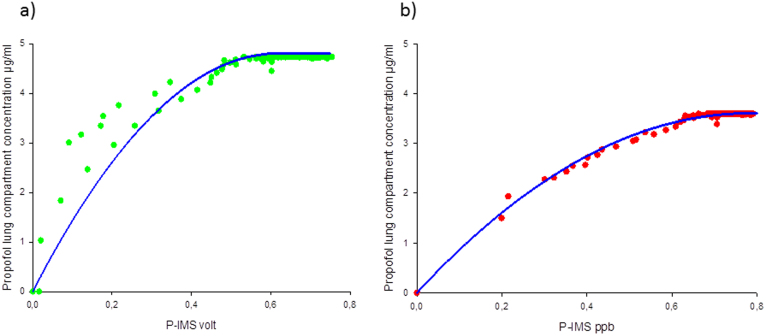
Propofol lung compartment concentration. The dots are the measured propofol concentration (a) green dots: peak height [volt]), (b) red dots: concentration [ppb] versus the propofol lung compartment concentration in µg/ml. The blue line is the polynomial second order.

**Figure 7:**
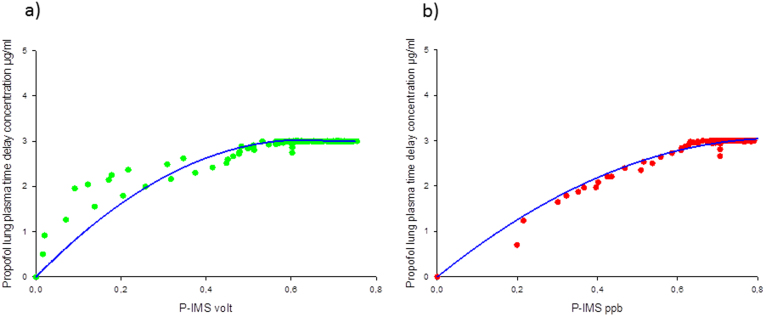
Time delay of the propofol plasma concentration. The dots are the measured propofol concentration (a) green dots: peak height [V], (b) red dots: concentration [ppb] versus the propofol lung plasma time delay concentration in µg/ml. The blue line is the polynomial second order.

The hysteresis loop collapsed by introduction of the propofol lung compartment concentration or a time delay of the propofol plasma concentration (Fig. 6/Fig. 7).

Using the measured breath concentration in ppb the propofol lung compartment of this individual patient could be described by a volume of 17 L and a clearance of 3,75 L/min. The rate constants were k1L = 0,209 min^−1^ and kL1 = 0,138 min^−1^. Using these parameters for calculation the lung compartment concentration yielded into a correlation coefficient of R^2^ = 0.99 (Fig. 6b). The polynomial second order is given by:

y = −5.9*x^2^ + 9.23*x + 0,

where y is the lung compartment concentration and x the propofol breath concentration.

The correlation value is lower using the original intensity values given in volt (R^2^ = 0.87)(Fig. 6a).

y = −12.6*x^2^ + 15.57*x + 0,

where y is the lung compartment concentration and x the propofol peak intensity.

### Time delay of the propofol plasma concentration

The time delay pharmacokinetic modeling approach results into a lower correlation coefficient R^2^ = 0.96 than using an additional compartment for describing the lung propofol concentration (Fig. 7).

The correlation between the model and measurements in ppb is R^2^ = 0.96 (Fig. 7b).

y = −4.2*x^2^ + 7.14*x + 0,

where y is the lung compartment concentration and x the propofol concentration.

The propofol lung plasma time delay concentration of this individual patient was calculated with a first- order rate constant determining the equilibration between plasma and lung concentration k_e0lung_ = 0.27 min^−1^.

The correlation value is lower using the original intensity values given as peak height measured in volt (R^2^ = 0.82) (Fig. 7a).

y = −7.7*x^2^ + 9.64*x + 0,

where y is the lung compartment concentration and x the propofol peak intensity.

## Discussion

In this manuscript two different pharmacological models are presented for the description of the pharmacokinetic of exhaled drug concentrations. The calculations were exemplarily performed for propofol, an intravenous anaesthetic widely used in clinical practice. The exhaled propofol concentration was measured with a modified MCC/IMS (P-IMS).

Under clinical conditions it is not possible to measure the propofol blood concentration online. Therefore, computer-assisted infusion pumps (Target controlled infusion (TCI)) were developed to calculate the propofol blood concentration based on the demographic patient data and the dosage^12^. A 3-compartment pharmacokinetic model is used for the calculation of the blood concentration. This blood or plasma concentration is given in the first compartment V1. Our first PK modeling approach is to extend this 3-compartment model for another 2-compartment model. The first compartment V1 is used parallel for both models. The second compartment of the 2-compartment model represents the lung compartment. The lung compartment has a volume (VLung). The volume is given in liters (L). The value is a virtual volume of distribution and not physiologically. The second parameter is the clearance of the compartment given in L/min (ClLung). Both values are used to calculate rate constants for metabolism and elimination. We have named these constants as k1L and kL1. kL1 is the deviation of the volume of the lung compartment and clearance of the lung compartment. k1L is required to balance the drug concentration between the lung compartment and the blood compartment. This PK approach has to be used if the drug is metabolized by the lung or if the drug accumulates in the lung. If there a metabolism of the drug in the lung the clearance value is positive. The clearance value is negative, if an accumulation is present. For propofol there are some publications from animal studies, hypothesizing that propofol is metabolized by the lung^16^.

Our second approach could be used, if there is only a time delay between changes of the drug concentration and exhaled concentrations. This PK model excludes changes of the concentration by the lung itself. The equilibration between plasma and lung concentration is described by a first-order rate constant (k_e0_). The k_e0_ value describes the time delay between changes of the blood concentration of anaesthetics and their effect at the brain measured with the EEG^17^. The transition of anaesthetics across the blood–brain barrier needs a certain time. The effects of anaesthetics on the brain can be measured with an EEG monitor^18^.

Then this method was transferred to describe the time delay between changes of the measured end-tidal concentration of volatile anaesthetics and the EEG effect^19^. Equilibration between end-tidal and brain concentrations of volatile anaesthetics is reached after a time delay that is substance-specific and called hysteresis, i.e. the delay between the maximum plasma concentration and the maximum EEG effect. In PK/PD models this hysteresis is considered by inclusion of a fictitious compartment, the so called “effect compartment”.

We used this mathematical procedure now to describe a time delay between changes of the drug blood concentrations and the exhaled concentrations. To distinguish between the “traditional” k_e0_ value^10^ and the new approach we introduced the term k_e0lung_. In generally, the higher the value of k_e0lung_, the shorter the time of equilibration between exhaled and blood concentration.

In our sample calculation for propofol both methods were suitable to describe the pharmacokinetic of the exhaled propofol concentration.

We used a polynomial of second order as a function for the PK model. For other drugs or biomarkers a linear correlation analysis could be sufficient. In other cases it could be necessary to choose a polynomial of a higher degree or a different mathematical model.

The calculations were performed for one individual patient. For one patient PK modeling could be done within Excel using the solver tool to optimize the correlation between the pharmacology model and measurements. For further analysis data of several patients in one step a Bayesian probability calculation is necessary^19^. The presented PK modeling methods are suitable for the continuous application of a drug. In principle they could also be used for a single bolus application.

The pharmacological principles demonstrated for propofol in this manuscript could be transferred to other medications. For propofol, further studies including measured propofol blood concentrations are necessary to build a PK model, which could be transferred into clinical practice.

Online measurement of drug concentrations in patient's breath is a new promising approach for individualized dosage of medications. Unfortunately there is no direct correlation between exhaled and blood concentration of the drug. Therefore pharmacokinetic modelling is necessary for research and clinical practice.

## Author Contributions

S.K., T.V. and J.I.B. wrote the manuscript. S.K. developed the pharmacokinetic models. S.K., S.M., A.H. prepared the excel sheet for the calculation. T.F. performed the patient measurement and prepared figures 1–3. All authors reviewed the manuscript.

## Figures and Tables

**Table 1:** Marsh data set^14^ (m is the weight of the patient expressed in kg)

V1	0,228*m [kg]
k10	0,1190
k12	0,1140
k21	0,0550
k13	0,0419
k31	0,0033
